# Supplementary material for: The Incidence of Sport-Related Anterior Cruciate Ligament Injuries: An Overview of Systematic Reviews Including 51 Meta-Analyses
Source: J Funct Morphol Kinesiol. 2025 May 14;10(2):174. doi: 10.3390/jfmk10020174 (PMC12101161; doi:10.3390/jfmk10020174)
Supplement: Supplementary file 1 [file jfmk-10-00174-s001.zip › Suppl File S1 Review protocol.pdf]

Supplementary file S1. Review protocol.

Study Information

Hypotheses

The prevalence and/or incidence of ACL is high in both sports and non-sport populations.

Design Plan

Study type

Other

Blinding

No blinding is involved in this study.

Is there any additional blinding in this study?

No response

Study design

An overview of systematic reviews.

No files selected

Randomization

No response

Sampling Plan

Existing Data

Registration prior to creation of data

Explanation of existing data

No response

Data collection procedures

Search strategy: The CINAHL, Embase, PubMed, and SPORTDiscus databases will be searched. Manual searches in those overviews of systematic reviews and review protocols related to the topic of this study will be checked if they appear during the study selection process. Eligibility criteria: The Population (including animal species), Exposure, Comparator, and Outcomes (PECO) framework will be followed. Morgan RL, Whaley P, Thayer KA, Schünemann HJ. Identifying the PECO: A framework for formulating good questions to explore the association of environmental and other exposures with health outcomes. Environ Int. 2018 Dec;121(Pt 1):1027-1031. doi:

10.1016/j.envint.2018.07.015. P = Sports and non-sport populations with anterior cruciate ligament injuries. No restrictions will be imposed in terms of age, gender, setting, or time since cancer diagnosis. E = not applicable. C = not applicable. O = the prevalence/incidence rates of anterior cruciate ligament injuries. Study design = systematic reviews with meta-analyses. We will follow the definition of systematic reviews proposed by the preferred reporting items for overviews of reviews (PRIOR) statement. Gates M, Gates A, Pieper D, Fernandes RM, Tricco AC, Moher D, Brennan SE, Li T, Pollock M, Lunny C, Sepúlveda D, McKenzie JE, Scott SD, Robinson KA, Matthias K, Bougioukas KI, Fusar-Poli P, Whiting P, Moss SJ, Hartling L. Reporting guideline for overviews of reviews of healthcare interventions: development of the PRIOR statement. BMJ. 2022 Aug 9;378:e070849. doi: 10.1136/bmj-2022-070849. Study selection: One reviewer will remove duplicates using bibliographic management software. Afterward, the same reviewer will read titles and abstracts and subsequently, will evaluate full texts when abstracts will seem eligible, or when abstracts will not be unavailable. Data extraction: one reviewer will extract information of interest from each systematic review when possible. For example, the number of studies and the total sample size of each meta-analysis of interest, the risk of bias assessment tool, or pooled prevalence/incidence rates of each outcome of interest.

No files selected

Sample size

Not applicable.

Sample size rationale

No response

Stopping rule

No response

Variables

Manipulated variables

No response

No files selected

Measured variables

Pooled prevalence and/or incidence rates of anterior cruciate ligament injuries in sports and/or non-sports populations.

No files selected

Indices

No response

No files selected

Analysis Plan

Statistical models

The methodological quality of systematic reviews: AMSTAR 2 will be used. The overlap among systematic reviews: matrices of evidence and the corrected covered area (CCA) will be applied to calculate the overlap. The overlap will be also visually represented. Maps of prevalence will be developed when possible.

No files selected

Transformations

No response

Inference criteria

No response

Data exclusion

No response

Missing data

No response

Exploratory analysis

No response

Other

Other

No response
