# Supplementary material for: The Incidence of Sport-Related Anterior Cruciate Ligament Injuries: An Overview of Systematic Reviews Including 51 Meta-Analyses
Source: J Funct Morphol Kinesiol. 2025 May 14;10(2):174. doi: 10.3390/jfmk10020174 (PMC12101161; doi:10.3390/jfmk10020174)
Supplement: Supplementary file 1 [file jfmk-10-00174-s001.zip › Suppl File S2 Search strategies.pdf]

Supplementary file S2. Search Strategies**.**

CINAHL (date 17/10/2023)

TX (ACL OR anterior-cruciate OR MH "Anterior Cruciate Ligament") AND AB (prevalence OR prevalent OR incidence OR epidemiology) AND TI (systematic-review OR meta-analysis OR meta-review OR meta-analytic-review OR metaanalysis OR meta-analyses)

Search modes - Boolean/Phrase.

Search filter: publication type: academic publications AND journals.

Search filter: language: English OR Spanish OR multiple languages.

Studies retrieved = 118

Embase (date 17/10/2023)

(ACL OR 'anterior cruciate':ti OR 'anterior cruciate ligament'/exp) AND (prevalence:ab,ti OR prevalent:ab,ti OR incidence:ab,ti OR epidemiology:ab,ti) AND ('systematic review':ti OR 'meta analysis':ti OR 'meta review':ti OR 'meta analytic review':ti OR metaanalysis:ti OR 'meta analyses':ti)

Search filter: publication type (review OR article OR article in press OR short survey).

Search filter: language: English OR Spanish.

Studies retrieved = 178

PubMed (date 17/10/2023)

(ACL [all] OR anterior-cruciate [all] OR Anterior Cruciate Ligament [mh]) AND (prevalence [tiab] OR prevalent [tiab] OR incidence [tiab] OR epidemiology [tiab]) AND (systematic-review [title] OR meta-analysis [title] OR meta-review [title] OR meta-analytic-review [title] OR metaanalysis [title] OR meta-analyses [title])

Search filter: language: English OR Spanish.

Studies retrieved = 223

SPORTDiscus (date 17/10/2023)

TX (ACL OR anterior-cruciate OR DE "ANTERIOR cruciate ligament") AND AB (prevalence OR prevalent OR incidence OR epidemiology) AND TI (systematic-review OR meta-analysis OR meta-review OR meta-analytic-review OR metaanalysis OR meta-analyses)

Search modes - Boolean/Phrase.

Search filter: publication type: academic publications AND journals.

Search filter: language: English OR Spanish.

Studies retrieved = 118
